# Impaired synaptic clustering of postsynaptic density proteins and altered signal transmission in hippocampal neurons, and disrupted learning behavior in PDZ1 and PDZ2 ligand binding-deficient PSD-95 knockin mice

**DOI:** 10.1186/1756-6606-5-43

**Published:** 2012-12-26

**Authors:** Hitoshi Nagura, Yasuyuki Ishikawa, Katsunori Kobayashi, Keizo Takao, Tomo Tanaka, Kouki Nishikawa, Hideki Tamura, Sadao Shiosaka, Hidenori Suzuki, Tsuyoshi Miyakawa, Yoshinori Fujiyoshi, Tomoko Doi

**Affiliations:** 1 Department of Biophysics, Graduate School of Science, Kyoto University, Kyoto 606-8502, Japan; 2 Cellular and Structural Physiology Institute, Nagoya University, Furo-cho, Chikusa, Nagoya, 464-8601, Japan; 3 Laboratory of Functional Neuroscience, Nara Institute of Science and Technology, Ikoma, Nara, 630-0192, Japan; 4 Department of Pharmacology, Graduate School of Medicine, Nippon Medical School, Tokyo, 113-8602, Japan; 5 Section of Behavior Analysis, Center for Genetic Analysis of Behavior, National Institute for Physiological Sciences, Okazaki, Aichi, 444-8585, Japan; 6 Cellular and Structural Physiology Institute, Nagoya University, Furo-cho, chikusa, Nagoya, 464-8601, Japan; 7 Division of Systems Medical Science, Institute for Comprehensive Medical Science, Fujita Health University, Toyoake, Aichi, 470-1192, Japan

**Keywords:** PSD-MAGUK, Synaptic clustering, PDZ domain, PSD-95, Synaptic transmission, Dentate gyrus, Behavioral test battery

## Abstract

**Background:**

Postsynaptic density (PSD)-95-like membrane-associated guanylate kinases (PSD-MAGUKs) are scaffold proteins in PSDs that cluster signaling molecules near NMDA receptors. PSD-MAGUKs share a common domain structure, including three PDZ (PDZ1/2/3) domains in their N-terminus. While multiple domains enable the PSD-MAGUKs to bind various ligands, the contribution of each PDZ domain to synaptic organization and function is not fully understood. Here, we focused on the PDZ1/2 domains of PSD-95 that bind NMDA-type receptors, and studied the specific roles of the ligand binding of these domains in the assembly of PSD proteins, synaptic properties of hippocampal neurons, and behavior, using ligand binding-deficient PSD-95 cDNA knockin (KI) mice.

**Results:**

The KI mice showed decreased accumulation of mutant PSD-95, PSD-93 and AMPA receptor subunits in the PSD fraction of the hippocampus. In the hippocampal CA1 region of young KI mice, basal synaptic efficacy was reduced and long-term potentiation (LTP) was enhanced with intact long-term depression. In adult KI mice, there was no significant change in the magnitude of LTP in CA1, but robustly enhanced LTP was induced at the medial perforant path-dentate gyrus synapses, suggesting that PSD-95 has an age- and subregion-dependent role. In a battery of behavioral tests, KI mice showed markedly abnormal anxiety-like behavior, impaired spatial reference and working memory, and impaired remote memory and pattern separation in fear conditioning test.

**Conclusions:**

These findings reveal that PSD-95 including its ligand binding of the PDZ1/2 domains controls the synaptic clustering of PSD-MAGUKs and AMPA receptors, which may have an essential role in regulating hippocampal synaptic transmission, plasticity, and hippocampus-dependent behavior.

## Background

Excitatory synapses in the mammalian brain are specialized by dense thickenings of proteins, referred to as postsynaptic densities (PSDs), in which glutamate receptors, cell adhesion proteins, scaffold proteins, and signaling molecules tightly associate via protein-protein interactions [1,2]. Two types of ionotropic glutamate receptors, AMPA-type (AMPAR) and NMDA-type (NMDAR), mediate excitatory synaptic transmission. PSD-95-like membrane-associated guanylate kinases (PSD-MAGUKs), including PSD-95, SAP102, PSD-93, and SAP97, are major scaffold proteins in the PSD [3]. These proteins comprise five protein-protein interacting domains, three PDZ domains in the N-terminus, followed by an src homology-3 (SH3) domain and a guanylate kinase (GK) domain in the C-terminus, and are thus thought to regulate synaptic transmission. The first and second PDZ (PDZ1/2) domains of PSD-95 bind to the extreme C-terminus of NMDAR subunit 2 (GluN2) and also interact with the C-termini of auxiliary subunits of AMPAR, transmembrane AMPAR regulatory proteins (TARPs), by which PSD-MAGUKs regulate AMPAR clustering at synaptic sites [4-6].

PSD-95 is a core component of the PSD. Based on quantitative mass spectroscopy, PSD-95 is ~6-fold more abundant than PSD-93, ~8-fold more than SAP102, and ~40-fold more than SAP97 in PSDs of the adult rat forebrain [7]. Therefore, PSD-95 is a key molecule in mature synapses. The results of a number of studies by means of acute knockdown of endogenous PSD-95 or knockout (KO) mice of PSD-95 suggest that PSD-95 has a critical role in regulating the subunit composition of NMDARs and the level of AMPARs and its activity-dependent change at synaptic sites via interactions with PDZ domains [8-14]. Further, the SH3 and GK domains in the C-terminus are essential for targeting PSD-95 to synaptic sites and the induction of NMDAR-dependent long-term depression (LTD) [15]. Due to functional redundancy among PSD-MAGUKs and their multiple protein-interacting domain structure, however, the specific roles of individual PSD-MAGUKs and each PDZ domain during development in vivo have remained unclear.

Here, we focused on the PDZ1/2 domains and generated mutant cDNA knockin (KI) mice in which the PDZ1/2 domains of PSD-95 were unable to bind ligands, but retained their overall structure, to assess its function in vivo with minimal uncontrollable compensatory effects by other PSD-MAGUKs [16,17]. We studied the developmental accumulation of PSD proteins, hippocampal synaptic transmission, and behavior of the KI mice. The KI mice showed decreased levels of mutant PSD-95, PSD-93, and AMPAR subunits, but increased levels of SAP102 in the PSD fraction; greatly enhanced hippocampal long-term potentiation (LTP); and abnormal anxiety-like behavior and deficits in spatial and conditioned fear memory. These findings suggest that PSD-95 plays a central role among PSD-MAGUKs in modulating synaptic functions.

## Results

### Generation of 1d2d-PSD-95-EGFP KI mice

**Figure 1:**
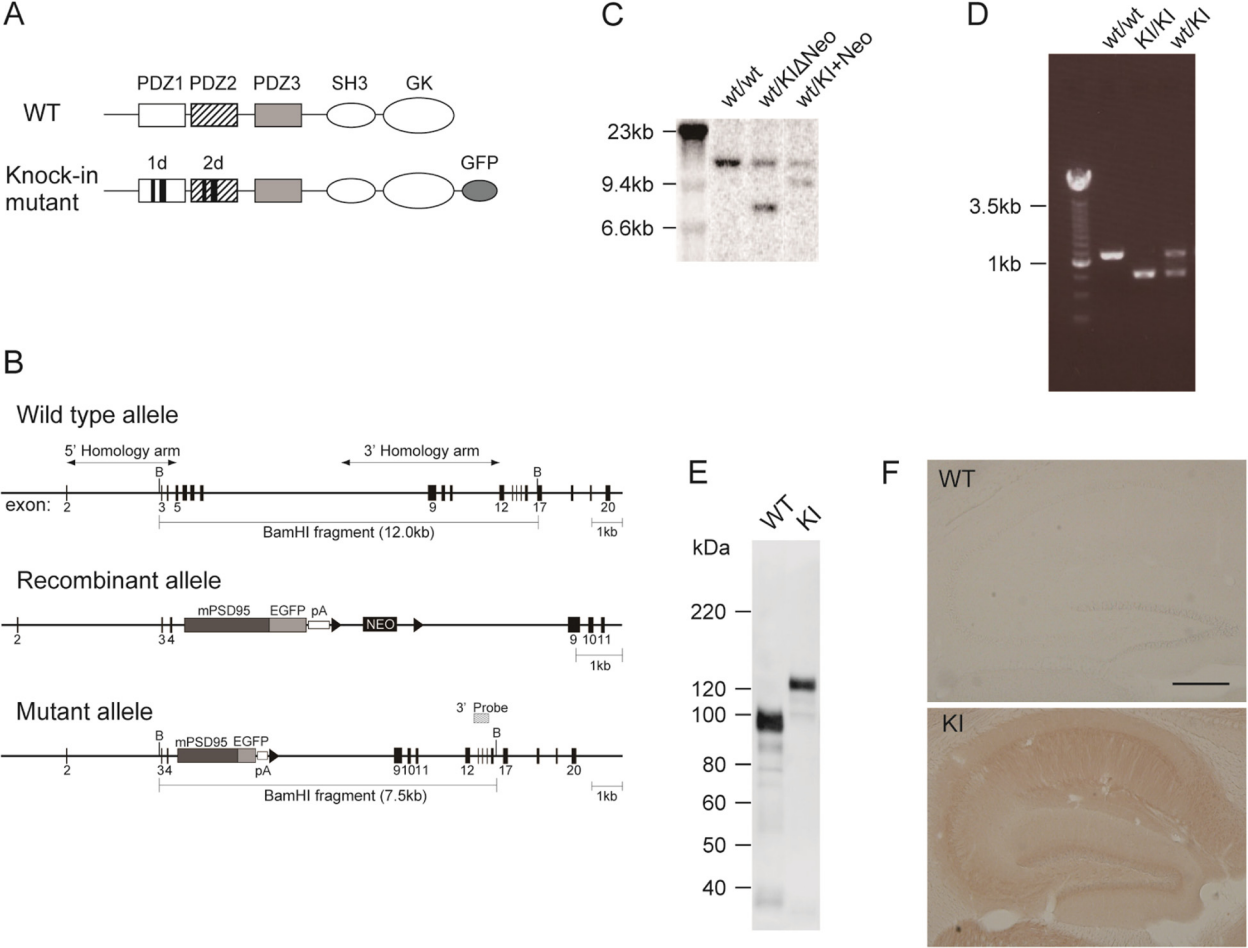
**Generation of the 1d2d-PSD-95-EGFP knockin mouse. A**, Domain structure of PSD-95. The mutant 1d2d-PSD-95-EGFP contains mutations in the PDZ1/2 domains and EGFP at the C-terminus, as described in the Materials and Methods. **B**, Schematic diagram of the PSD-95 gene locus, the recombinant allele after homologous recombination, and the mutant allele after Cre recombination. The region from exon 5 to the middle of intron 8 was replaced by the corresponding region of mutant PSD-95 cDNA connected with the EGFP gene (see methods). B, BamHI. **C**. Southern blot analyses of mouse genomic DNA to confirm homologous recombination. Mouse genomic DNA was digested with BamHI and hybridized with a 3’ external probe, whose position is indicated in **B**. wt/wt, wild-type mouse; wt/KIΔNeo, heterozygous KI mouse after deleting the Neo cassette; wt/KI+Neo, heterozygous KI mouse containing the Neo cassette. **D**. Genotyping of the mouse by PCR amplification of the region from exon 4 to exon 8 to detect an ~300-bp size shift. wt/wt, wild-type mouse; KI/KI, homozygous KI mouse; wt/KI, heterozygous KI mouse. **E**, Immunoblot of brain extracts from wild-type (WT) and homozygous KI (KI) mice. The whole brain crude membrane fractions from WT (2 μg total protein) and KI (20 μg total protein) mice were analyzed with an anti-PSD-95 K28/43 antibody. **F**, Immunohistochemical staining of sagittal sections from the hippocampus of WT and homozygous KI adult mice with an anti-GFP antibody. Extensive staining was observed in the hippocampal fields of the KI mice, but not in the WT mice. Scale bar = 500 μm.

To investigate the specific roles of the PDZ1 and PDZ2 domains of PSD-95 among PSD-MAGUK proteins in the assembly of PSD proteins, we introduced a mutated PSD-95 cDNA coding for ligand-binding deficient PDZ1 and PDZ2 domains followed by the PDZ3-SH3-GK domains and fused with EGFP (1d2d-PSD-95-EGFP) into exon 5, which encodes the PDZ1 domain, as described in the Materials and Methods (Figure 1A, B). The four targeted ES lines were identified by Southern blotting (Figure 1C) and used to generate germ-line transmitted KI mice. Interbreeding of KI heterozygous mice yielded offspring at the expected Mendelian ratio. Genotyping was performed by PCR (Figure 1D). Homozygous 1d2d-PSD-95-EGFP KI mice were noticeably smaller than the WT mice around 4 to 8 weeks of age. Crossing 1d2d-PSD-95-EGFP KI homozygotes rarely generated offspring; moreover, the homozygous female mice did not nurture their pups.

To confirm expression of the 1d2d-PSD-95-EGFP gene in KI mice, brain extracts from WT and mutant mice were analyzed by immunoblotting with an anti-PSD-95 antibody (Figure 1E). While the WT brain extracts showed a ~95-kDa major band for WT PSD-95, the KI brain extracts showed a ~125-kDa major band, i.e., mutant PSD-95 fused with EGFP. Immunohistochemistry using an anti-GFP antibody also showed expression of GFP fusion protein in all of the hippocampal fields of KI mice, suggesting expression of the 1d2d-PSD-95-EGFP gene (Figure 1F). Electron microscopy observations revealed no drastic ultrastructural abnormalities in our histologic evaluation of adult KI mouse brains (Additional file 1: Figure S1).

### Altered composition of PSD proteins in the hippocampus of 1d2d-PSD-95-EGFP KI mice during development

PSD-95 was initially identified as an abundant protein in the PSD fraction and plays important roles in clustering receptors, other scaffolding proteins, and signaling molecules by interactions via its N-terminal region three PDZ domains, and SH3 and GK domains. The 0.5% Triton X100-insoluble PSD fraction is considered to reflect an assembly of postsynaptic density proteins [7,18]. The 1d2d-PSD-95-EGFP expressed in hippocampal neurons was localized to excitatory postsynaptic sites, although loss of the ligand-binding ability of PDZ1/2 domains caused its inefficient synaptic clustering, ~50% that of WT [19]. Therefore, we first confirmed enrichment of 1d2d-PSD-95-EGFP in the PSD fraction of whole brains by subcellular fractionation. The synaptic localization of 1d2d-PSD-95-EGFP was confirmed by the PSD-95 signals colocalized with an excitatory presynaptic marker, vesicular glutamate transporter-1, in hippocampal cultures from KI mice (Additional file 1: Figure S2).

**Figure 2:**
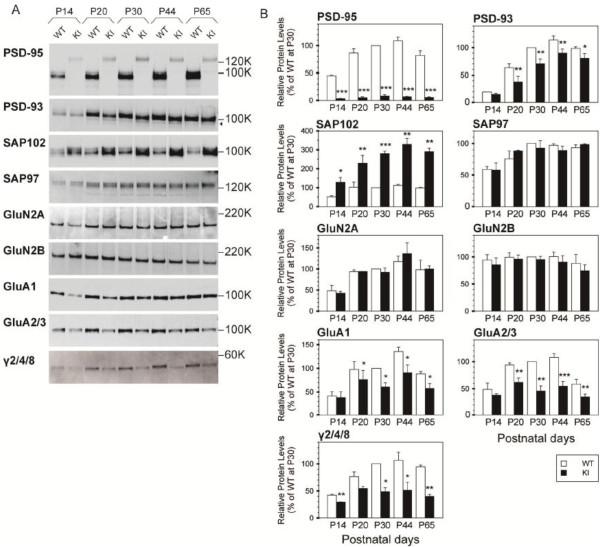
**Altered PSD protein composition in the hippocampal PSD fraction of KI mice during development. A**, Immunoblot analyses of PSD fractions from WT and KI mice at the respective developmental ages for the indicated proteins. The following amounts of WT and KI PSD proteins were analyzed per lane: PSD-93, 0.5 μg; SAP102, 0.5 μg; SAP97, 2 μg; GluN2A, 0.5 μg; GluN2B, 0.5 μg; GluA1, 0.5 μg; GluA2/3, 0.5 μg; and γ2/4/8, 2 μg. The arrowhead indicates the degraded bands of PSD-93. For PSD-95, 0.25 μg of WT and 0.5 μg of KI PSD proteins were analyzed for comparison on identical membrane filters and their band intensities were corrected in **B**. **B**, Quantitation of various proteins in the PSD fractions based on the results shown in **A**, normalized to the wild-type level at P30 (set as 100%). The histograms show the mean ± SEM (open bars, WT; filled bars, KI). Three sets of PSD fractions at various ages (P14–P65) were examined. Simple comparison of the means and SEM of WT and KI data was performed using Student’s t-test. *P < 0.05, **P < 0.005, ***P < 0.001. In the KI PSD fraction, extensively low levels of mutant PSD-95, significantly decreased levels of PSD-93, GluA1, GluA2/3, and γ2/4/8, and increased levels of SAP102 during development are observed compared to the WT mice.

We then compared the amounts of PSD-MAGUK proteins in the PSD fractions prepared from the hippocampi of 1d2d-PSD-95-EGFP KI mice at P14, P20, P30, P44, and P65 with those from WT littermates. While the developmental accumulation of 1d2d-PSD-95-EGFP in the PSD fraction was similar to that of the WT protein, the amounts contained in a defined amount of protein were significantly lower, i.e., 8.1 ± 2.4% of the amount observed in WT mice at P30 (Figure 2B). In contrast, the accumulation of SAP102 in the PSD fractions was remarkably elevated in the KI mice, approximately 3-fold higher than in the WT mice at P20–P65 (280.6 ± 11.9% at P30), whose time course of expression was consistent with that of PSD-95 [20,21], suggesting that the ligand-binding ability of the PDZ1/2 domains of PSD-95 was largely displaced by SAP102. Unexpectedly, another PSD-MAGUK protein, PSD-93, showed reduced PSD accumulation at P20–P65, particularly significant at P20–P30, with approximately 60% to 70% of the levels observed in the WT mice (70.0 ± 9.1% at P30). The low molecular-weight signals under the PSD-93 band (arrowhead in Figure 2A) observed simultaneously might be due to loss of the ligand-binding ability of the PDZ1/2 domains of PSD-95, leading to destabilization of PSD-93 in the PSD fraction, presumably via its association with 1d2d-PSD-95-EGFP. Levels of the last member of the PSD-MAGUK proteins, SAP97, were not affected at any age. Thus, all of the PSD-MAGUK proteins, i.e., SAP102, PSD-93, and SAP97, accumulated differentially in the PSD fractions of KI mouse hippocampus in accordance with the expression of 1d2d-PSD-95-EGFP.

Studies of PSD-95 KO mice and knockdown by short hairpin RNA indicate that PSD-95 is required for NMDAR and AMPAR clustering at synaptic sites [9,10,12]. Therefore, we examined the amounts of NMDAR subunits (GluN2A, and GluN2B) and AMPAR subunits (GluA1, GluA2/3, and γ2/4/8 TARPs) in the hippocampal PSD fraction of KI mice. TARPs are auxiliary subunits of AMPARs, which are also known as calcium channel γ-subunit homologs, and are the primary PDZ ligands of PSD-95, of which γ8 TARP is mainly expressed in the hippocampus [22]. GluN2A and GluN2B signals in the PSD fraction of KI mice from P14–P65 were similar to those of WT mice, except that the GluN2A signal at P44 in the KI mice tended to be larger than that in their WT littermates. Conversely, γ2/4/8 TARPs, GluA1, and GluA2/3 signals were significantly reduced during development. Decreases of γ2/4/8 TARPs and GluA2/3 in the PSD fractions became gradually distinct from P14–P20 in the KI mice and their levels were ~50% those in the WT mice from P30–P65 (48.6 ± 7.3%, 45.7 ± 9.4% at P30, respectively). Coincidentally, the level of GluA1 in the PSD fractions of KI mice was reduced to 60% to 70% (60.7 ± 8.9% at P30) those in the WT mice.

The reduced levels of PSD-93, GluA1, and GluA2/3 in the PSD fractions of KI mice became less significant at P65 compared to those at P30, and at even older ages, the differences between the WT and KI PSD fractions became smaller.

Increased expression of SAP102 and decreased expression of 1d2d-PSD-95-EGFP and PSD-93 in the hippocampus of 1d2d-PSD-95-EGFP KI mice

**Figure 3:**
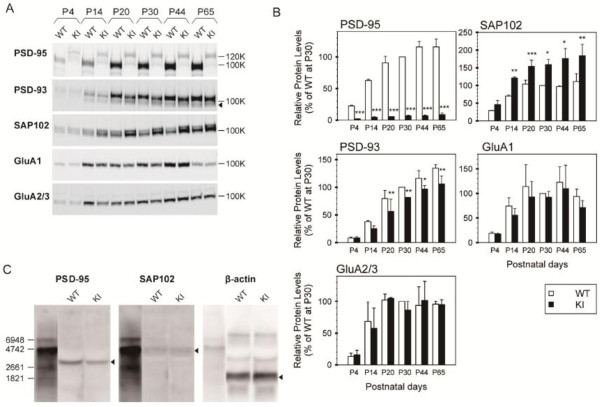
**Altered expression of SAP102 and PSD-93 in the hippocampus of KI mice. A**, Immunoblot analyses of hippocampal homogenates from WT and KI mice at the respective developmental ages for the indicated proteins. The following amounts of homogenate proteins were analyzed per lane: PSD-93 and SAP102, WT and KI: 5 μg; GluA1 and GluA2/3, WT and KI: 10 μg; and PSD-95, WT: 5 μg, KI: 20 μg. The arrowhead indicates the degraded bands of PSD-93. **B**, Quantitation of PSD proteins in the hippocampal homogenates based on the results shown in **A**, normalized to the wild-type levels at P30 (set as 100%). The histograms show the mean ± SEM (open bars, WT; filled bars, KI). Three sets of hippocampal homogenates at various ages (P4–P65) were examined. The means and SEM of WT and KI data were compared using Student’s t-test. *P < 0.05, **P < 0.005, ***P < 0.001. In the KI PSD homogenates, extensively low levels of mutant PSD-95, significantly decreased levels of PSD-93, and increased levels of SAP102 were observed during development compared to the WT mice, while the levels of GluA1 and GluA2/3 were not significantly different from those of the WT mice. **C**, Northern blot analyses of PSD-95 and SAP102 mRNA. Ten micrograms of total RNA prepared from WT and KI mice hippocampi were analyzed in a formaldehyde-containing 1% agarose gel. The numbers on the left side indicate the sizes of the digoxigenin-labeled RNA molecular weight markers. Arrowheads indicate the major band for each mRNA.

Because PSD-MAGUKs accumulated differently in the PSD fraction of the KI mice compared to their WT littermates, we analyzed homogenates of hippocampal neurons from P4–P65 to examine if the differences were due to their expression or trafficking to synaptic sites. While the protein levels of 1d2d-PSD-95-EGFP in the homogenates were one-tenth to one-twentieth the levels observed in the WT mice from P4–P65 (7.1 ± 0.8% at P30), the levels of SAP102 were increased by 1.5- to 2-fold those in WT mice (159 ± 15% at P30, Figure 3A, B). Conversely, band intensities of PSD-93 from P14–P65 were significantly decreased (with a gradual increase of lower bands during development) in KI mice to ~80% (81.5 ± 0.3% at P30) the level observed in WT mice. In contrast, GluA1 and GluA2/3 expression was not significantly different between the KI and WT mice.

Because the expression of 1d2d-PSD-95-EGFP and SAP102 was largely altered in the hippocampal homogenates, we analyzed mRNA from the hippocampi of WT and KI mice to examine whether the observed changes were induced during transcription or at/after translation. Northern blotting showed that the expression of 1d2d-PSD-95-EGFP mRNA was approximately 50% of that in WT mice, whereas there was no significant difference in the expression of SAP102 mRNA between the KI and WT mice (Figure 3C). Therefore, SAP102 expression may be increased after the transcriptional step in KI mice. Considering that the 50% level of 1d2d-PSD-95-EGFP mRNA was expressed, compared to that of the WT, extensive protein degradation and/or inefficient synaptic clustering of 1d2d-PSD-95-EGFP after its expression might occur in vivo, probably due to mutations and the lack of ligand-binding ability of the PDZ1/2 domains. It is also possible that the reduced level of 1d2d-PSD-95-EGFP mRNA could be due to the gene targeting strategy of knocking in a partial cDNA. The bands under the intact PSD-93 bands in the homogenates from KI mice appeared to be more intense than those from the WT. The band intensity gradually increased during development in concert with the reduced levels of intact PSD-93 in the homogenates from KI mice. Therefore, the lower bands were likely to be degraded PSD-93 and its degradation might partly correspond to its reduced levels in the PSD fractions from KI mice (Figure 2). In addition, the unchanged expression of GluA1 and GluA2/3 in KI mice suggests that their reduced accumulation in the PSD fraction was not due to their expression, but rather to reduced synaptic targeting via the lack of binding to the auxiliary subunits of AMPAR, γ2/4/8, with the PDZ1/2 domains of PSD-95.

### Altered synaptic transmission and plasticity in the hippocampus of young adult 1d2d-PSD-95-EGFP KI mice

**Figure 4:**
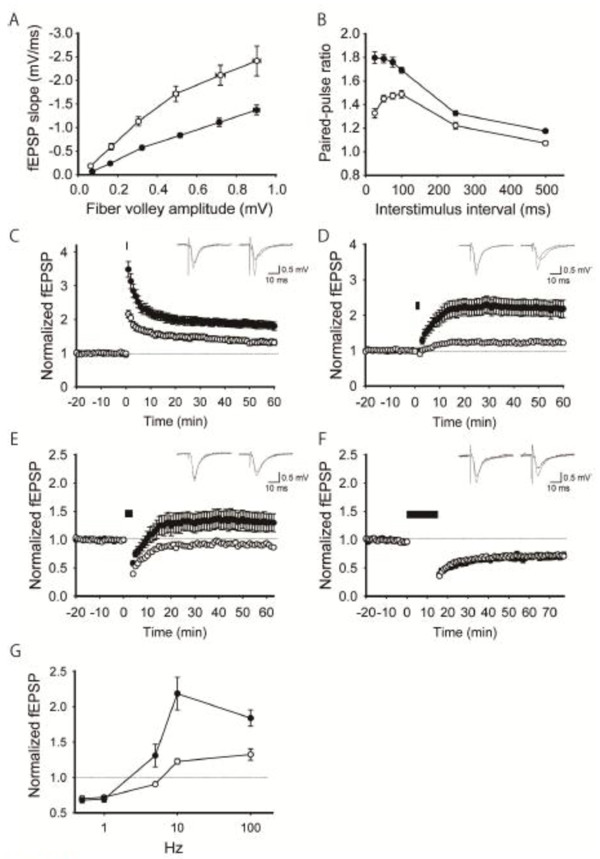
**Enhanced LTP and normal LTD in the hippocampal CA1 region of young adult 1d2d-PSD-95-EGFP KI mice. A**, The synaptic input–output relationship in slices from 6- to 8-week-old WT (open circles, n = 16 slices from 4 mice) and KI (filled circles, n = 12 slices from 4 mice) mice. **B**, Paired pulse facilitation plotted as a function of the interstimulus interval. Filled circles: KI mice (n = 12 slices from 4 mice). Open circles: WT littermates (n = 16 slices from 4 mice). **C**, LTP by high-frequency stimulation (100 Hz, 100 pulses). Filled circles: KI mice (n = 8 slices from 3 mice). Open circles: WT littermates (n = 7 slices from 3 mice). **D**, LTP by intermediate-frequency stimulation (10 Hz, 900 pulses). Filled circles: KI mice (n = 10 slices from 4 mice). Open circles: WT littermates (n = 6 slices from 4 mice). **E**, LTP by low-frequency stimulation (5 Hz, 900 pulses). Filled circles: KI mice (n =7 slices from 5 mice). Open circles: WT littermates (n = 6 slices from 4 mice). **F**, LTD by low-frequency stimulation (1 Hz, 900 pulses). Filled circles: KI mice (n = 6 slices from 3 mice). Open circles: WT littermates (n = 8 slices from 3 mice). Bars in C-F indicate the period of high/low frequency stimulation. **G**, Summary of synaptic plasticity at different stimulation intensities in slices from WT (open circles) and KI (filled circles) mice. All values are expressed as mean ± SEM.

PSD-95 has critical roles in synaptic functions through NMDAR signaling [8-11]. To explore the roles of the ligand binding of the PDZ1/2 domains of PSD-95 in basal synaptic transmission, we first examined the input/output relationship at Schaffer collaterals/commissural projections-CA1 synapses in acute hippocampal slices from 6- to 8-week-old WT and 1d2d-PSD-95-EGFP KI mice. The ratios between the amplitude of the presynaptic fiber volley (input) and the initial slope of postsynaptic fEPSPs (output) were reduced by ~50% over a range of stimulus strengths in slices from 1d2d-PSD-95-EGFP KI mice relative to those of WT controls (Figure 4A). This result suggests that basal AMPAR-mediated synaptic transmission is reduced to ~50% in slices from 1d2d-PSD-95-EGFP KI mice, which coincides with the reduced levels of GluA1 and GluA2 in the hippocampal PSD fraction (Figure 2). We next recorded the paired-pulse ratio (PPR), which is inversely related to the presynaptic release probability [23]. The PPR at all interstimulus intervals tested was significantly larger in slices from 1d2d-PSD-95-EGFP KI mice than from WT mice, suggesting that the probability of neurotransmitter release was also reduced at these synapses in the mutant mice (Figure 4B).

We then investigated NMDAR-dependent long-term synaptic plasticity in the CA1 region (Figure 4C–F). A high-frequency stimulation protocol (100 pulses at 100 Hz) induced robust LTP in both WT and mutant slices, and the magnitude of LTP was significantly larger in slices from 1d2d-PSD-95-EGFP KI mice than in WT slices (WT: 132.4 ± 8.0%, n = 7; KI: 183.9 ± 11.6%, n = 8 at 60 min after tetanus, Figure 4C). An intermediate-frequency stimulation (900 pulses at 10 Hz) also induced enhanced LTP in slices from KI mice (WT: 122.6 ± 3.8%, n = 6; KI: 218.6 ± 23.0%, n = 10, Figure 4D). Even a lower-frequency stimulation at 5 Hz with 900 pulses, close to the threshold for LTP induction in WT mice (90.4 ± 2.4%, n = 6), potentiated fEPSPs to 130.9 ± 16.0% (n = 7) that of baseline in KI mice (Figure 4E). In contrast, LTD evoked by low-frequency stimulation (1 Hz, 900 pulses) was indistinguishable between the WT (71.9 ± 2.7%, n = 8) and 1d2d-PSD-95-EGFP KI (69.4 ± 4.4%, n = 6) mice (Figure 4F). As summarized in Figure 4G, the potentiation level and frequency dependence of LTP induction in the CA1 region of the hippocampus were altered in slices from 6- to 8-week-old 1d2d-PSD-95-EGFP KI mice, whereas the magnitude of LTD was unchanged compared to slices from WT mice. Thus, the loss of ligand-binding ability of the PDZ1/2 domains of PSD-95 and its decreased level resulted in reduced basal synaptic transmission of AMPAR, increased paired-pulse facilitation, and altered long-term synaptic plasticity.

### Synaptic plasticity in the hippocampus of older 1d2d-PSD-95-EGFP KI mice

**Figure 5:**
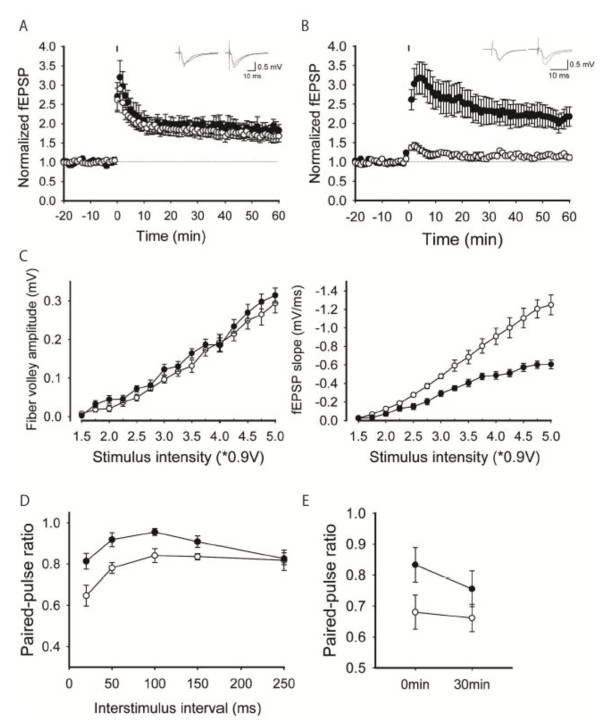
**Synaptic properties of hippocampal slices from older (>10 weeks) WT and 1d2d-PSD-95-EGFP KI mice. A**, LTP by high-frequency stimulation (100 Hz, 100 pulses) at Schaffer collaterals/commissural projections-CA1 synapse in WT (open circles, n = 7 slices from 3 mice) and KI (filled circles, n = 6 slices from 3 mice) mice. **B**, LTP by repeated tetanus stimulation in the continuous presence of picrotoxin (100 μM) at medial perforant path-DG synapses. Filled circles: KI mice (n = 8 slices from 4 mice). Open circles: WT littermates (n = 8 slices from 4 mice). Bars in A,B indicate the period of single/repeated high frequency stimulations. **C**, Basal transmission of medial perforant path-DG synapses. Filled circles: KI mice (n = 8 slices from 3 mice). Open circles: WT littermates (n = 8 slices from 3 mice). Fiber volley amplitude was plotted against the stimulus intensity in the left panel, and the initial fEPSP slope was plotted against the stimulus intensity in the right panel. **D**, PPR of the medial perforant path-DG synapses. Filled circles: KI mice (n = 5 slices from 3 mice). Open circles: WT littermates (n = 5 slices from 3 mice). **E**, PPR with 50 ms interstimulus interval of the medial perforant path-DG synapses of WT (open circles, n = 8 slices from 4 mice) and KI (filled circles, n = 10 slices from 4 mice) mice was measured before and after LTP induction. All values are expressed as mean ± SEM.

Next, we further examined the synaptic plasticity at the same synapses in older mice usually used for behavioral studies (>10 weeks old). In contrast with the slices from young mice, LTP levels in the slices from older 1d2d-PSD-95-EGFP KI and WT mice induced by the high-frequency stimulus were indistinguishable (WT: 167.9 ± 15.1%, n = 7; KI: 182.6 ± 15.6%, n = 6, at 60 min after tetanus Figure 5A). This trend of developmental change was also seen with mild tetanic stimulation (900pulses of 5Hz), whereas the reduced basal transmission remained at older age (data not shown). We then tested whether the lack of changes in LTP is commonly observed at other excitatory synapses on principal neurons in the hippocampus of 1d2d-PSD-95-EGFP KI mice. We focused on medial perforant path-DG synapses, which also exhibit NMDAR-dependent LTP, as a comparison with Schaffer collaterals/commissural projections-CA1 synapses. In our experimental condition, repeated tetanic stimulation induced weak LTP at the medial perforant path-DG synapses in the adult WT mice (114.1 ± 7.9%, n = 8, Figure 5B). Unexpectedly, the same repeated tetanic stimulus could induce remarkably larger LTP in slices from adult 1d2d-PSD-95-EGFP KI mice than in slices from WT mice (223.9 ± 26.5%, n = 8, Figure 5B). The robust LTP at the medial perforant path-DG synapses was also expressed in young mice, and the amplitude was indistinguishable with that in old mice (WT: 112.5 ± 12.5%, n = 7, KI: 209.1 ± 15.8%, n = 8; Additional file 1: Figure S4). Analogous with the Schaffer collaterals/commissural projections-CA1 synapses of young 1d2d-PSD-95-EGFP KI mice, basal transmission of the medial perforant path-DG synapses was attenuated to ~50% in older 1d2d-PSD-95-EGFP KI mice (Figure 5C). The decreased release probability of neurotransmitters was also suggested by the increased PPR in 1d2d-PSD-95-EGFP KI mice (Figure 5D). This raises the possibility that the tetanic stimulation not only induced normal NMDAR-dependent LTP, but also rescued presynaptic suppression in KI mice, thereby causing strongly enhanced synaptic potentiation. To test whether altered presynaptic properties are related to increased LTP, we measured PPR before and at 30 min after LTP induction. We observed no significant change, however, between these ratios in 1d2d-PSD-95-EGFP KI mice (84.2 ± 5.1% and 74.9 ± 5.2% of PPR before and at 30 min after LTP induction in KI mice, n = 10, *P* = 0.102, Figure 5E), suggesting the lack of presynaptic mechanisms for LTP expression. This result indicates that a presynaptic mechanism does not explain the increased level of LTP at DG synapses.

### Hypoactivity and abnormal anxiety-like behavior of 1d2d-PSD-95-EGFP KI mice in a novel environment

To evaluate the behavioral effects of PSD-95 deficiency, we subjected 1d2d-PSD-95-EGFP KI mice and their WT littermates to a comprehensive battery of behavioral tests. The 1d2d-PSD-95-EGFP KI mice weighed ~10% less than their WT littermates (Table 1; genotype effect, F_(1,30)_ = 15.49; *P* = 0.0005) and showed slightly higher grip strength (genotype effect, F_(1,30)_ = 4.47; *P* = 0.043), but no significant differences in body temperature (genotype effect, F_(1,30)_ = 1.26; *P* = 0.270), sensory-motor gating (prepulse inhibition; genotype effect, F_(1,30)_ = 0.13; *P* = 0.718 at 110 dB, F_(1,30)_ = 0.18; *P* = 0.672 at 120 dB ), pain sensitivity (hot plate test; genotype effect, F_(1,30)_ = 0.26; *P* = 0.616), and motor coordination (accelerating rotarod tests; genotype effect, F_(1,30)_ = 2.57; *P* = 0.1193). On the other hand, acoustic startle responses were significantly reduced (genotype effect, F_(1,30)_ = 21.84; *P* < 0.0001) and latency to fall in wire hang test was shorter in KI mice (genotype effect, F_(1,30)_ = 4.52; *P* = 0.0418).

**Table 1:** General physical characteristics and sensory/motor functions of 1d2d-PSD-95-EGFP KI mice and their wild-type littermates

	**WT**	**KI**
Physical characteristics		
Weight (grams)	28.5 (± 0.8)	24.9 (± 0.5)
Rectal temperature (°C)	37.1 (± 0.2)	36.9 (± 0.2)
Grip strength	0.86 (± 0.019)	0.93 (± 0.029)
Sensory motor reflex		
Acoustic startle response (arbitrary unit)		
Stimulus intensity = 110 dB	0.9 (± 0.07)	0.5 (± 0.09)
Stimulus intensity = 120 dB	1.1 (± 0.07)	0.6 (± 0.08)
Prepulse inhibition (%; startle stimulus = 110 dB)		
Prepulse intensity = 74 dB	32.1 (± 5.2)	38.2 (± 10.2)
Prepulse intensity = 78 dB	58.4 (± 4.0)	44.9 (± 9.7)
Pain test		
Hot plate (latency; seconds)	9.2 (± 0.6)	8.9 (± 0.4)
Motor tests		
Wire hang (latency to fall; seconds)	56.4 (± 1.9)	43.6 (±5.8)
Rotarod (latency to fall; averages of three trials)		
Day 1	118.8 (± 21.0)	89.6 (± 16.2)
Day 2	183.3 (± 14.9)	151.3 (± 18.0)

Data represent the mean (±SEM) (WT mice, n = 16, KI mice, n = 16).

**Figure 6:**
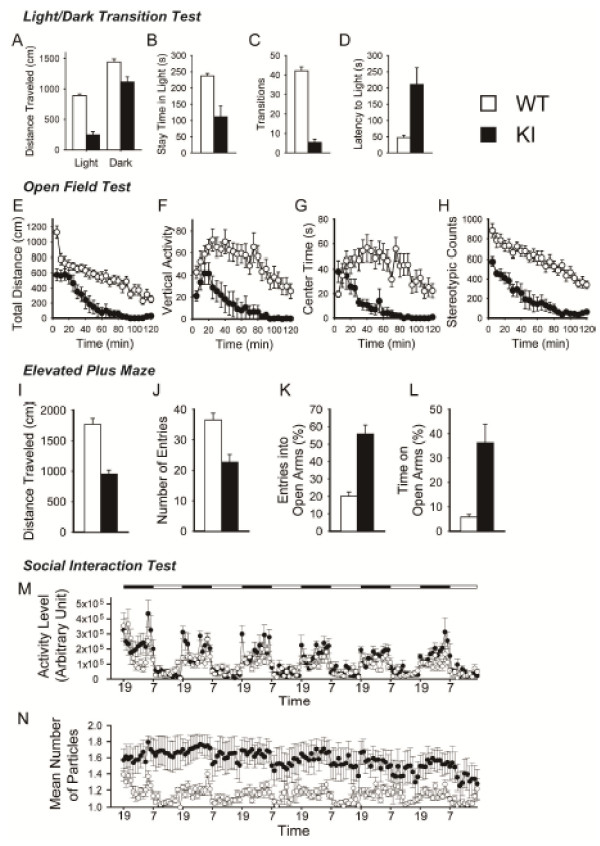
**Hypoactivity and increased anxiety-related behavior in a novel environment and reduced social interactions in the home cages of 1d2d-PSD-95-EGFP KI mice. A–D**, Light/dark transition test (WT controls, open bars, n = 15; KI, filled bars, n = 16): distance traveled in the light/dark compartments (**A**), time spent in the light compartment (**B**), number of light/dark transitions (**C**), and latency to enter the light compartment (**D**) were recorded. **E–H**, Open field test (WT controls, open circles, n = 16; KI, filled circles, n = 16): total distance traveled (**E**), vertical activity (**F**), time spent in the center of the compartment (**G**), and stereotypic counts (**H**) were recorded. **I–J**, Elevated plus maze test (WT controls, open bars, n = 15; KI, filled bars, n = 13): distance traveled in the arms (**I**), number of entries into the arms (**J**), number of entries into the open arms (**K**), and time spent in the open arms (**L**) were recorded. **M–N**, Social interaction test in the home cages (WT controls, open circles, n = 7; KI, filled circles, n = 6): KI mice were more active at night than WT mice (**M**) and the mean number of particles representing the mice acting separately were significantly higher all day long in the KI mice compared to the WT mice (**N**).

The 1d2d-PSD-95-EGFP KI mice were significantly less active in a novel environment than their WT littermates, as measured by distance traveled in the light/dark transition test (Figure 6A; WT: 889.4 ± 27.0 cm; KI: 245.9 ± 56.1 cm; genotype effect, F_(1,29)_ = 102.34; *P* < 0.0001 in the light, WT: 1442.6 ± 48.4 cm; KI: 1115.9 ± 81.8 cm; genotype effect, F_(1,29)_ = 11.43; *P* = 0.0021 in the dark, one-way ANOVA), total distance (Figure 6E, genotype effect, F_(1,30)_ = 30.1; *P* < 0.0001) and vertical activity (Figure 6F, genotype effect, F_(1,30)_ = 18.9; *P* = 0.0001) in the open field test, distance traveled (Figure 6I, WT: 1771.0 ± 95.9 cm; KI: 951.9 ± 65.9 cm; genotype effect, F_(1,26)_ = 46.59; *P* < 0.0001, one-way ANOVA ), and total number of arm entries in the elevated plus maze (Figure 6J, WT: 36.5 ± 2.3; KI: 22.6 ± 2.6; genotype effect, F_(1,26)_ = 16.20; *P* = 0.0004, one-way ANOVA). The KI mice also exhibited markedly reduced repetitive stereotypic behavior in the open field (Figure 6H, genotype effect, F_(1,30)_ = 40.6; *P* < 0.0001). Further, the significant hypoactivity of KI mice in novel environments was also observed in the Y-maze test (see Additional file 1: Figure S1).

In addition, the 1d2d-PSD-95-EGFP KI mice showed significantly increased anxiety-like behavior, as revealed by their shorter stay time in the light (Figure 6B, WT: 237.4 ± 7.9 s; KI: 110.8 ± 34.3 s; genotype effect, F_(1,29)_ = 12.17; *P* = 0.0016, one-way ANOVA), fewer transitions between compartments (Figure 6C, WT: 42.2 ± 2.0 ; KI: 5.4 ± 1.6; genotype effect, F_(1,29)_ = 209.2; *P* < 0.0001, one-way ANOVA), and longer latencies to enter the light side (Figure 6D, WT: 47.7 ± 7.0 s; KI: 211.1 ± 51.0 s; genotype effect, F_(1,29)_ = 9.43; *P* = 0.0046, one-way ANOVA) in the light/dark transition test. Further, the mutant mice spent significantly less time in the center zone of the open field (Figure 6G, genotype effect, F_(1,30)_ = 27.8; *P* < 0.0001), which is considered to reflect increased anxiety.

In the elevated plus maze test, the mutant mice entered the open arms more frequently (Figure 6K, WT: 20.1 ± 2.4%; KI: 55.9 ± 5.1%; genotype effect, F_(1,26)_ = 43.63; *P* < 0.0001, one-way ANOVA), and spent more time in the open arms (Figure 6L, WT: 5.8 ± 1.1%; KI: 36.3 ± 7.6%; genotype effect, F_(1,26)_ = 18.16; *P* = 0.0002, one-way ANOVA), which is usually considered a sign of decreased anxiety-like behavior. However, increased time spent in the open arms is sometimes interpreted as “panic-like escaping behavior” in mice showing increased anxiety-like behavior in other tests [24,25]. Therefore, the observed behavioral abnormalities may reflect heightened anxiety by the mutant mice subjected to an unfamiliar environment in the elevated plus maze test.

### Social interaction is normal in a novel environment but decreased in their home cages

In the social interaction test conducted in a novel environment, the total duration of contacts, number of contacts, total duration of active contacts, mean duration per contact did not differ between genotypes (genotype effect, F_(1,14)_ = 0.12, *P* = 0.733; F_(1,14)_ = 0.072, *P* = 0.793; F_(1,14)_ = 0.23, *P* = 0.639; F_(1,14)_ = 0.977, *P* = 0.340; see Additional file 1: Figure S5), while distance traveled was significantly lower in the KI mice (genotype effect, F_(1,14)_ = 39.35, *P* < 0.0001), possibly due to its hypoactivity. On the other hand, in social interaction tests in their home cages under familiar conditions, the KI mice were more active at night than their WT littermates (Figure 6M, genotype effect, F_(1,11)_ = 8.1; *P* = 0.02 at night) and the time spent separately was significantly higher all day long (Figure 6N, genotype effect, F_(1,11)_ = 10.5; *P* = 0.008), suggesting remarkably lowered social interactions in the mutant mice. This result also suggests that the hypoactivity of the KI mice observed above in the novel environment in the light/dark transition test and open field test was not due to a general decrease in motor activity. In the three-chamber social interaction test, KI mice showed a significantly reduced stay time around the cages, suggesting decreased interest in other mice, in addition to their hypoactivity in the test context (Additional file 1: Figure S5). These behaviors might correspond to autism-related disorders.

### Impaired spatial learning and memory in the 1d2d-PSD-95-EGFP mice

**Figure 7:**
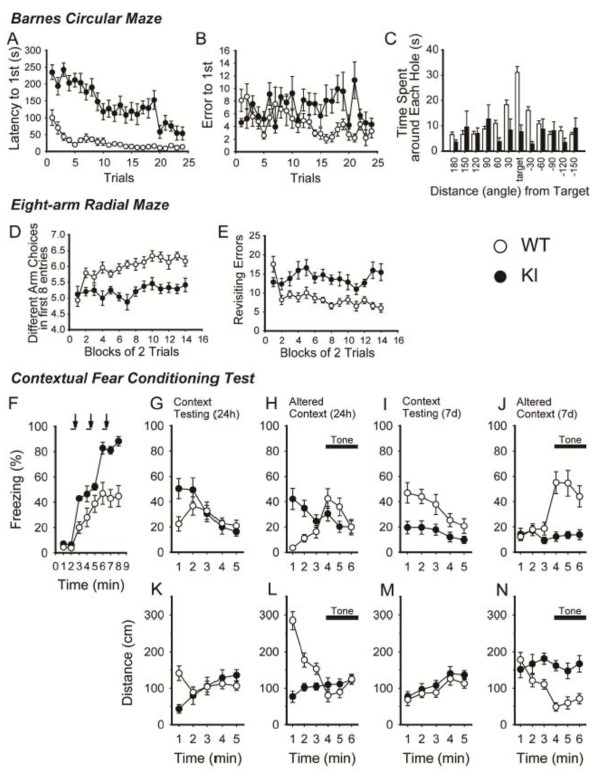
**Impaired spatial learning and fear memory in the 1d2d-PSD-95-EGFP KI mice. A–C**, Barnes circular maze (WT, open circles, n = 15; KI, filled circles, n = 16): latency to reach the target hole (**A**) and number of errors before reaching the target hole (**B**) across the training sessions were counted. Time spent around each hole in the probe test conducted after 24 trials, as shown in **A** and **B** was recorded (**C**). **D–E**, Eight-arm radial maze (WT controls, open circles, n = 15; KI, filled circles, n = 12): KI mice showed low levels of different arm choices in the first 8 entries (**D**), and increased revisiting errors (**E**) during training. **F–N**, Contextual fear conditioning test. In the fear conditioning phase, KI mice (filled circles, n = 12) showed higher freezing responses than WT controls (open circles, n = 15) (**F**). Horizontal bars denote exposure to the conditioning stimulus (tone), and arrows indicate the timing of the unconditioned stimulus (footshock). Freezing behaviors and distance traveled in the context test at 24 h after fear conditioning (**G, K**). Freezing behaviors and distance traveled in the cued test with an altered context at 24 h after fear conditioning (**H, L**). Freezing behaviors and distance traveled in the context test at 7 days after fear conditioning (**I, M**). Freezing behaviors and distance traveled in the cued test with altered context at 7 days after fear conditioning (**J, N**).

In the Barnes circular maze, spatial learning based on distal environmental room cues was evaluated by taking advantage of the ability of mice to find and escape through small holes into a dark box. The KI mice showed a significantly longer latency to find the target hole when the dark box was recessed under the platform (Figure 7A, genotype effect, F_(1,29)_ = 50.3; *P* < 0.0001), and their number of errors before finding the target hole was significantly increased (Figure 7B, genotype effect, F_(1,29)_ = 35.7; *P* < 0.0001). To confirm learning, the probe test, in which the escape box was removed, was performed at 24 h after trial #24 (3 trials per day for 8 successive days). While the WT littermates spent the longest time around the target hole, the KI mice showed no preference for the target hole (Figure 7C, WT: 31.1 ± 2.3 s; KI: 7.7 ± 2.6 s; *P* < 0.0001). The results of the probe test suggest that the 1d2d-PSD-95-EGFP KI mice have severely impaired spatial reference memory.

In the 8-arm radial maze test, the mice explored 8 arms baited with food at the end of each arm. The number of different arm choices among the first 8 entries, which is considered to reflect working memory, revealed a significantly lower number in the KI mice (Figure 7D, genotype effect, F_(1,25)_ = 73.7; *P* < 0.0001). Similarly, the KI mice displayed a significantly increased number of revisiting errors, in which the mice returned more than once to the same arm where the food had already been obtained (Figure 7E, genotype effect, F_(1,25)_ = 62.0; *P* < 0.0001). These observations suggest that the 1d2d-PSD-95-EGFP KI mice have impaired spatial working memory.

### Impaired fear memory in the 1d2d-PSD-95-EGFP KI mice

To measure the ability of the mice to learn and remember an association between an aversive experience and environmental cues, the KI mice and their WT littermates were subjected to contextual fear conditioning in which long-term memory can be established in a single conditioning trial. During the conditioning period, KI mice showed higher levels of freezing in response to the tone-shock pairs (Figure 7F, genotype effect, F_(1,25)_ = 17.6; *P* = 0.0003). This high freezing level might be due to the increased level of anxiety in KI mice, but also indicates acquisition of the fear response. The distance traveled during the 4-s interval after the initiation of footshocks was similar between WT and KI mice (data not shown). The latency in a pain test using a hot plate of the KI mice was also similar to that of the WT mice (Table 1), suggesting the sensitivity to footshock in the KI mice was comparable to that of the WT mice. In the context test conducted at 24 h after conditioning, the KI mice showed significantly higher freezing during the first minute (Figure 7G, freezing, WT: 22.4 ± 5.7%; KI: 50.3 ± 7.9%; *P* = 0.007; distance traveled, WT: 141.0 ± 19.9 cm; KI: 43.0 ± 11.7 cm during the first minute). Similarly, in the cued testing with altered context at 24 h after conditioning, higher freezing by the KI mice was observed only during the first 1 to 2 min, followed by the same level of freezing observed in the WT mice in response to the conditioned stimulus (tone, Figure 7H, WT: 3.7 ± 1.0% ; KI: 42.2 ± 8.3%; *P* = 0.0004; distance traveled, WT: 284.7 ± 24.3 cm; KI: 76.5 ± 15.8 cm; *P* < 0.0001 during the first minute). The distance travelled well reflected the freezing ratios. These observations suggest that KI mice have deficits in contextual discrimination or pattern separation, in addition to an increased level of anxiety.

Further, in the context testing and cued testing with an altered context one week after conditioning, the KI mice showed significantly less freezing (Figure 7I, genotype effect, F_(1,25)_ = 12.2, *P* = 0.0018) and complete unresponsiveness to the tone, with the distance traveled being xunaffected by the tone (Figure 7J, freezing, WT: 55.0 ± 8.6% ; KI: 12.4 ± 3.7%; *P* = 0.0009; distance traveled, WT: 48.3 ± 11.4 cm; KI: 161.7 ± 13.9 cm; *P* < 0.0001 at the 4th minute). These data suggest either altered persistence of fear memory, or enhanced fear extinction in KI mice. Thus, the 1d2d-PSD-95-EGFP KI mice exhibited deficits in the process of fear memory acquisition, consolidation, and retention for at least 7 days, which is thought to be mediated mainly through the amygdala, hippocampus, and medial prefrontal cortex.

## Discussion

The present study, using mutant PSD-95 cDNA KI mice, showed that PSD-95 is essential for the normal assembly of PSD-MAGUKs and the assembly of at least ~50% of basal AMPAR levels in the PSD, normal induction of synaptic plasticity in the hippocampus, and normal hippocampus-dependent behavior, such as learning and memory.

The genetically modified KI mice presented here expressed 1d2d-PSD-95-EGFP at ~7% of the level of WT PSD-95 in hippocampal homogenates. PSD-95 and other PSD-MAGUKs also interact with non-PDZ ligands, such as SAPAP/GKAP proteins via its GK domains, which in turn interact with another family of scaffolding proteins, Shank, and microtubule-associating motor protein dynein [26-28]. Altered PSD-MAGUK protein levels might also change protein association contexts at synapses via their interaction with these molecules. Therefore, the observed phenotypes could be attributable to the substantial reduction in the levels of PSD-95, which interact with PDZ ligands as well as non-PDZ ligands, the expression of PDZ1/2-domain ligand binding-deficient PSD-95, and/or compensation by other PSD-MAGUKs. A number of the properties of the KI mice, however, were surprisingly different from those of PSD-95 KO mice, e.g., reduced levels of PSD-93 and AMPAR subunits in PSDs during development, normal magnitude of LTD induction, noticeably small body weight, marked hypokinetics in an unfamiliar environment, and markedly abnormal anxiety-related behavior. These different properties are considered to be the result of the expression of 1d2d-PSD-95-EGFP in vivo, although the possibility of different compensatory effects in KI mice from those in KO mice could not be excluded. Thus, the targeted introduction of mutant PSD-95 into mice allowed us to have a better understanding of the specific roles of the ligand-binding of the PDZ1/2 domains of PSD-95 in synaptic functions.

Loss of the ligand-binding ability of the PDZ1/2 domains of PSD-95 led to altered levels of PSD-MAGUKs in the PSD fraction of the hippocampus, substantially low levels of mutant PSD-95 (~8% of that of WT at P30), significantly reduced levels of PSD-93 (~70% that of WT at P30), and increased accumulation of SAP102 (~280% of that of WT at P30) during development (Figure 2). This increased expression of SAP102 was also observed in hippocampal homogenates (Figure 3). Similarly, the increased expression of SAP102 in hippocampal extracts is reported in PSD-95 KO mice and PSD-95/PSD-93 double KO mice, suggesting a certain compensatory function of SAP102 [9,29]. On the other hand, decreased levels of PSD-93 in the PSD fraction are not observed in the PSD-95 KO mice. Because PSD-93 can form heteromultimers with PSD-95 in vivo and 1d2d-PSD-95-EGFP is unstable in spines, a fraction of PSD-93 directly interacting with 1d2d-PSD-95-EGFP might be degraded [30].

With regard to the assembly of glutamate receptors in hippocampal PSDs, a marked reduction (to ~50%) of AMPAR subunits, GluA1, GluA2, and TARPs, was observed from P20–P65 in KI mice, whereas the levels of the NMDAR subunits GluN2A and GluN2B in the PSD fraction and GluA1 and GluA2 in hippocampal homogenates from KI mice were not significantly affected (Figures 2, 3). These results suggest that the ligand-binding ability of the PDZ1/2 domains of PSD-95 or the PSD-95 protein level itself regulates synaptic targeting of at least ~50% of basal AMPARs via TARPs, but not of NMDARs in hippocampal neurons in vivo during development. In PSD-95 KO and PSD-95/PSD-93 double KO mice, the replacement of GluN2B-containing NMDARs with GluN2A-containing NMDARs is failed during synapse maturation [10,12]. The biochemical investigation of the hippocampal PSD fraction performed here may not have been sensitive enough to detect the replacement of GluN2B- with GluN2A-NMDARs.

The deficiency in PSD-95 also caused a reduction in basal AMPAR-mediated synaptic transmission, as determined by the reduced input/output ratios (Figure 4A, 5C), and enhanced the magnitude of LTP following high frequency stimulation in hippocampal Schaffer collaterals/commissural projections-CA1 and medial perforant path-DG synapses of KI mice. The reduced basal synaptic efficacy is consistent with the reduced levels of GluA1 and GluA2/3 in the PSD. Since PPR was increased in KI mice, presynaptic changes may also be involved in the reduced synaptic efficacy. In the PSD-95 KO mice, the enhanced LTP observed in the CA1 region was suggested to originate from an increase in the population of silent synapses, which are preferential sites of AMPAR insertion during LTP [10,11,31]. The enhanced LTP observed in the KI mice could be caused by the same mechanism. KI mice older than 10 weeks, however, did not show enhanced LTP in the CA1 region (Figure 5A), whereas basal transmission was still decreased at older age. Accumulation of the PSD proteins that compensate for the defects may explain this developmental change of LTP, and a presynaptic effect may be predominantly involved in the reduced basal transmission in older age. Further, LTP was enhanced at DG synapses in older mice (Figure 5B). These findings suggest that the ligand-binding ability of the PDZ1/2 domains of PSD-95 is differentially involved in mechanisms underlying the induction of NMDAR-dependent LTP in CA1 and DG.

The absence of LTD was previously observed in hippocampal slices from PSD-95 KO mice but not PSD-93 KO mice, and the importance of PSD-95 in the induction of LTD has been also demonstrated [8,11,15,32,33]. Moreover, studies using short hairpin RNA-mediated knockdown of endogenous PSD-95 or PSD-93 expression suggest considerable heterogeneity of excitatory synapses in CA1 pyramidal neurons with respect to the levels of PSD-95 and PSD-93 [9,34]. Taken together, these observations imply that the induction of LTD leads to decreases in the levels of APMARs only from synapses that mainly express PSD-95. In contrast to the increased levels of LTP, however, a normal level of LTD was still induced in hippocampal slices from KI mice following low frequency stimulation, despite the low expression level of 1d2d-PSD-95-EGFP. One possible explanation for the normal induction of LTD is that the impaired basal transmission is a result of the reduced AMPARs to nearly the same extent in both types of synapses that mainly express PSD-95 or PSD-93. Indeed, our KI mice had reduced amounts of not only PSD-95 but also PSD-93 (Figure 2B), and similar reduction of basal transmission with normal LTD is reported in TARP γ-8 KO mice as the consequence of general retraction of AMPAR from hippocampal synapses [35]. Another possibility is that a compensatory mechanism that was different from that of PSD-95 KO mice enabled the induction of LTD in the synapses that mainly express PSD-93. Thus, the normal induction of LTD observed here could be due to either reduced basal AMPAR transmission or an equal level of LTD induction at the synapses that mainly express PSD-93 and those that mainly express 1d2d-PSD-95-EGFP.

Despite the clustering of 1d2d-PSD-95-EGFP at postsynaptic sites, some presynaptic alterations were also suggested in the KI mice (Figsures 4B, 5D). Consistently, a similar observation was reported using PSD-95 KO or knockdown experiments [8,36]. Further, the relationships between PSD-MAGUKs and the presynaptic releaseprobability were suggested at other excitatory synapses [37]. The increased PPR could be at least partly due to the reduced levels of 1d2d-PSD-95-EGFP and the subsequent decreased levels of 1d2d-PSD-95-EGFP-neuroligin complexes. Considering the intact PDZ3 domain of our construct and the effectiveness of 1d2d-PSD-95-EGFP protein judged by normal LTD induction, however, the significance of PDZ1/2 domains that do not bind to neuroligin should be taken into account.

We performed comprehensive behavioral analyses of KI mice. These mice exhibited abnormal anxiety-related behavior, significant hypoactivity in novel environments, and decreased social interactions in their home cages. These behavioral abnormalities may be related to neurodevelopmental disorders, such as autism, via synaptic dysfunction caused by the loss of PSD-95 itself and/or the ligand-binding ability of the PDZ1/2 domains of PSD-95 [38]. Autism is characterized by abnormal social interactions, deficits in communication, and high levels of repetitive behaviors. In the three-chambered social interaction tests, KI mice showed a significant lack of interest in others based on their stay time around the cages, as well as decreased locomotor activity (Additional file 1: Figure S5). In the Y-maze test, KI mice showed remarkably decreased exploration and impaired working memory, as well as repetitive exploration of the same arm with few alternations. These behaviors might comprise autism-like features. On the other hand, markedly decreased stereotypic counts in the open field tests suggest that increased self-grooming by the KI mice is unlikely in the novel environment (Figure 6H). In PSD-95 KO mice, significantly increased self-grooming behaviors were observed only in the home cage [38]. Further, synaptic proteins, such as Shank and neuroligin, which interact with PSD-95, are also implicated in autism [39]. The relation of PSD-95 with autism should be studied further.

In the fear conditioning test, while KI mice showed higher freezing during fear conditioning and in the first 1 to 2 min in the cued testing with an altered context at 24 h after conditioning (Figure 7H), they showed a significantly decreased freezing response after 7 days in the altered context (Figure 7J). This trend in the freezing level was also observed with context testing (Figure 7G,I) Therefore, although we could not exclude the possibility that hypoactivity and a high anxiety level of KI mice affected the freezing measurement, high freezing behaviors observed in the KI mice were associated with aversive experiences, suggesting that KI mice are impaired in fear memory retention or enhanced fear extinction.

The observed severe deficits in spatial, working, and fear memory are likely to be associated with abnormal synaptic transmission in the hippocampus, particularly in DG synapses, because the behavioral tests were conducted in adult KI mice older than 3 months of age. Further studies to investigate the roles of PSD-95 at the medial perforant path-DG synapses will provide a better understanding of the contribution of DG synapses to learning and memory formation.

Our study shows that PSD-95 and/or the ligand-binding ability of the PDZ1/2 domains of PSD-95 are crucial for normal synaptic clustering of the PSD-MAGUKs and AMPAR subunits; normal synaptic transmission in the hippocampus; and normal learning and memory formation, including acquisition, consolidation and retention. The results encourage future studies, such as a detailed comparative analysis of PSD-95 KO mice and 1d2d-PSD-95-EGFP KI mice and overexpression studies of 1d2d-PSD-95-EGFP in vivo that will clarify the specific roles of the ligand-binding ability of the PDZ1/2 domains of PSD-95.

## Conclusions

In summary, our biochemical and electrophysiological analyses of 1d2d-PSD-95-EGFP KI mice indicate that PDZ1/2 domains of PSD-95 play crucial roles in postsynaptic density protein clustering, synaptic transmission and plasticity both in the hippocampal CA1 and DG with different age dependence. The battery of behavioral test also showed their significance in several hippocampus-dependent behaviors. Further studies are required to elucidate underlying molecular mechanisms which link the ligand binding deficiency of these domains and phenotypes of KI mice.

## Methods

### Generation of 1d2d-PSD-95 KI mice

The targeting vector for the generation of 1d2d-PSD-95 KI mice was constructed by amplifying a 5’ homology arm (~4.0 kb, the region from exon 2 to exon 5) and a 3’ homology arm (~5.1 kb, region from intron 8 to exon 12) from the PSD-95 genomic sequence (Ensemble gene ID: ENSMUSG00000020886) using mouse C57BL/6 J genomic DNA (Figure 1B). Following intron 4 of the endogenous PSD-95/Dlg4 gene, exon 5 was replaced with the 2.9-kb 1d2d-PSD-95-EGFP cDNA fragment coding from the PDZ1 to enhanced green fluorescent protein (EGFP) at the C-terminus, accompanied by a polyA tail derived from the pFGFP-C1 vector (BD Biosciences-Clontech, Palo Alto, CA, USA). 1d2d-PSD-95 contained ligand-binding deficient PDZ1/2 domains that were mutated to mimic the PDZ3 domain structure so that they retained, more or less, the PDZ domain structure [16,17]. These domains contained the mutations S78N (AGC to AAC), A80V (GCA to GTA), S183N (AGC to AAC), and A185V (GCA to GTA), and the deletions T83 to I88 and V178 to I183. EGFP was fused to the C-terminus of the mutated PSD-95 via a GGGSS linker [19], so that the KI mutants were easily identified by the molecular-weight difference between PSD-95 and 1d2d-PSD-95-EGFP (Figure 1A). The expression of 1d2d-PSD-95-EGFP in hippocampal neurons has been shown to target postsynaptic sites [19]. A 2-kb neomycin resistance cassette (Neo) flanked with two loxP sites was inserted downstream of the PSD-95 gene in the same transcriptional orientation. Embryonic stem cell (ES) lines carrying the mutated PSD-95-EGFP-Neo gene (wt/KI+Neo) were generated by homologous recombination in Bruce4 C57BL/6 J ES cells (OZ gene) and identified by Southern blot hybridization (Figure 1B, C). Four clones were injected independently into BALB/c blastocysts to generate chimeras that transmitted the mutation into the germ line. To eliminate the Neo cassette, coat color chimeras were crossed with C57BL/6 J mice that carry the cre transgene under the control of the PGK promoter at the ROSA26 locus and express Cre recombinase in early embryonic stages (OZ gene). Heterozygous mice carrying the 1d2d-PSD-95-EGFP gene (wt/KIΔNeo, Figure 1C) were subsequently backcrossed onto the C57BL/6 J strain to remove the cre transgene and bred under specific-pathogen free conditions. Homozygous mice carrying the 1d2d-PSD-95-EGFP gene were obtained by mating heterozygous littermates and analyzed by polymerase chain reaction (PCR) genotyping using primers for the exon 4 antisense strand and exon 8 sense strand (Figure 1D). The Bruce4 C57BL/6 J ES cell DNA sample varies at 34 markers (12.4%) from C57BL/6 J DNA among 275 simple sequence length polymorphisms tested [40]. DNA from homozygous 1d2d-PSD-95-EGFP or littermate wild-type (WT) mice showed variations at 2.5 of those 34 markers in an average of 2 mice. Further, analysis of 200 single nucleotide polymorphisms that discriminate C57BL/6 J and 129 strains estimated the genetic background to be ~98% C57BL/6 J congenicity in an average of 2 mice, both in the homozygous 1d2d-PSD-95-EGFP and littermate WT mice. All animal experiments were performed according to the guidelines approved by the Animal Experimentation Committee of Kyoto University.

### Preparation of PSD fractions from mouse brain

PSD fractions were prepared from whole mouse brains or dissected hippocampi at various ages at 4°C [18,41-43]. Brains or dissected tissues were placed in ice-cold solution A (0.32 M sucrose, 1 mM NaHCO_3_, 1 mM MgCl_2_, 0.5 mM CaCl_2_, 0.1 mM phenylmethylsulfonyl fluoride, and a 1:1000-diluted protease inhibitor cocktail [Nacalai Tesque]), as 10% wet weight tissue/volume. Typically, hippocampi from two P14–P44 mouse brains, weighing 40 to 100 mg, were homogenized in 0.4 to 1.0 mL of buffer A using a Dounce homogenizer with 10 strokes. The homogenates were centrifuged at 1400 × g for 10 min and the supernatants saved. The pellet was rehomogenized in the same volume of buffer A and centrifuged at 710 × g for 10 min. The two supernatants were combined and further centrifuged at 13,800 × g for 15 min. The obtained crude membrane pellet (P2) was resuspended in 200 μL of solution B (0.32 M sucrose, 1 mM NaHCO_3_) and loaded onto a discontinuous sucrose gradient (1 mL each of 1 M/1.4 M sucrose solution in 1 mM NaHCO_3_), followed by centrifugation at 82,500 × g in a TLS55 rotor for 65 min. The synaptosomal fraction between 1 M and 1.4 M sucrose was collected, diluted 4-fold with 5 mM HEPES (pH 7.5), and centrifuged at 28,000 × g in a TLA100.3 rotor for 20 min. The recovered pellet was resuspended in 100 to 200 μL of 5 mM HEPES (pH 7.5), in which the protein concentration ranged from 0.1 to 0.3 mg/mL, and incubated with an equal volume of 1% Triton X-100, 5 mM HEPES (pH 7.5) solution for 15 min. The suspension was centrifuged at 50,000 × g in a TLA100.3 rotor for 20 min. The resulting pellet (PSD-I) was used as the PSD fraction. Because the first Triton X-100 extraction was performed at a relatively low protein concentration, the second Triton X-100 extraction showed no significant differences other than decreased recovery. Therefore, in the developmental studies of hippocampal PSD fractions, the second Triton X-100 treatment was omitted. For the P14, P20, P30, and P44 hippocampal PSD fractions, two mouse brains were dissected. For the P65 hippocampal PSD fraction, one mouse brain was used. The PSD fractions at each age were prepared independently three times. We recovered 40 to 120 μg of hippocampal PSD proteins from each P14–P65 brain.

### Immunoblot analyses and immunohistochemistry

The following antibodies were used: anti-PSD-95 (mouse monoclonal; K28/43; 1:1000; NeuroMab), anti-PSD-93 (rabbit polyclonal; 1:200; Alomone Labs), anti-SAP97 (mouse monoclonal; K64/15; 1:200; NeuroMab), anti-GluA1 (rabbit polyclonal; 1:400; Chemicon), anti-GluA2/3 (rabbit polyclonal; 1:500; Chemicon), anti-GluN2A (mouse monoclonal; 1:500; BD Transduction Laboratories), anti-GluN2B (mouse monoclonal; 1:250; BD Transduction Laboratories), anti-TARPGamma2/4/8 (mouse monoclonal; N245/36; 1:200; NeuroMab), and anti-SynGAP (rabbit polyclonal; 1:1000; Affinity BioReagents), anti-AKAP150 (rabbit polyclonal; 1:1000; Millipore), anti-pan-Shank (mouse monoclonal; 1:1000; StressMarq Biosciences), anti-mGluR5 (rabbit polyclonal; 1:2000; Millipore), anti-Homer antiserum (rabbit polyclonal; 1:10000). The rabbit polyclonal anti-SAP102 antibody was provided by Dr. J. H. Hell (University of Iowa). To compare the amounts of protein in the PSD fractions at various ages, quantitative immunoblot analyses were performed. The protein concentrations of the PSD fractions were determined using a BCA protein assay in the presence of 0.2% sodium dodecyl sulfate, according to the manufacturer’s protocol (Thermo Scientific). Depending upon the affinity of the first antibody, 0.5 to 5 μg of protein from each PSD fraction was analyzed by sodium dodecyl sulfate-polyacrylamide gel electrophoresis. After incubation with the horseradish peroxidase-conjugated secondary antibody, the blotted protein bands were visualized using an ECL detection kit (GE Healthcare), detected by an LAS-3000, and analyzed using Multi Gauge (Fuji Film) according to the manufacturer’s instructions. Immunoblotting was performed three times for one set of PSD fractions at various ages and three sets of PSD fractions at various ages were analyzed. The quantitated band intensities were normalized to the level in WT mice at P30 (set as 100, Figsures 2B, 3B) and expressed as means ± standard error of the mean (SEM). The statistical difference was determined using a two-tailed Student’s t test.

For immunostaining of brain tissue, 12-week-old WT and 1d2d-PSD-95-EGFP KI mice were anesthetized and perfused transcardially with 4% paraformaldehyde, as described previously [44]. Preparation of brain sections and immunostaining with anti-GFP antibody (rabbit polyclonal; 1:1000; Medical & Biological Laboratories) were also performed as described previously [44].

### Northern hybridization analyses

Total RNA was extracted from adult forebrains (10-week-old mice) using a phenol and guanidium thiocyanate method and analyzed by Northern blot hybridization using digoxigenin-labeled probes for PSD-95, SAP102, and β-actin, which were prepared by PCR, according to the manufacturer’s procedure (Roche). The band intensities were quantified using an LAS-3000 (Fuji Film). The ratios of mutant versus WT intensities for PSD-95 and SAP102 were corrected according to the level of β-actin (Figure 3C). The major lengths estimated from the genomic sequence for each mRNA were as follows: WT PSD-95, 3069 bp; KI PSD-95, 3138 bp; SAP102, 4958 bp; and β-actin, 1892 bp. The experiments were repeated twice.

### Electrophysiology

Field excitatory postsynaptic potentials (fEPSPs) were recorded in the CA1 region and the dentate gyrus (DG) of young (6–8 weeks) and old (>10 weeks) WT and 1d2d-PSD-95-EGFP KI mice. Preparation of hippocampal slices from young mice and the subsequent fEPSP recordings in the stratum radiatum of the CA1 region were performed as described previously [45]. Transverse slices (300 μm thick) from the mid-hippocampus of old mice were prepared using a tissue slicer (D.S.K. LINEARSLICER PRO7). The isolated hippocampus was cut in an ice-cold artificial cerebrospinal fluid (ACSF) composed of (in mM): NaCl, 125; KCl, 2.5; MgSO_4_, 1.3; CaCl_2_, 2.5; glucose, 11; NaH2PO_4_, 1; NaHCO_3_, 26.2, and continuously bubbled with 95% O_2_/5% CO_2_. The slices were incubated at 30°C for 30 min and then maintained in an interface chamber at room temperature (20–26°C) for at least 2 h before use.

Each slice was placed in a submersion-type recording chamber with ACSF flowing at 2 mL/min at 27°C. Picrotoxin (100 μM) was added to the ACSF when fEPSPs were recorded from the DG region to block GABA_A_ receptors. The recording pipette was filled with 2 M NaCl, and fEPSPs were evoked with a bipolar tungsten stimulating electrode. Stimulus was adjusted so that induced fEPSP do not reach the saturated level, and delivered at a frequency of 0.05 Hz. In each hippocampal subfield, over 20 min of baseline recordings were obtained prior to the induction of LTP. LTP was induced using a standard single tetanus protocol at the CA1 region, i.e., 1 s at 100 Hz, and using a repeated tetanus protocol at the DG region, i.e., 0.5 s at 100 Hz repeated 3 times at 20-s intervals. The fEPSP slope was measured at ~1-ms time windows positioned on the early rising phase. All of the recordings from the hippocampal slices of old mice were made using an HEKA EPC10 amplifier (HEKA Elektronik, Germany), filtered at 2 kHz, and digitized at 10 kHz. The data were stored in a personal computer for subsequent analysis. All values are expressed as the mean ± SEM. Statistical significance was evaluated using a two-tailed Student’s t-test or paired t-test with a significance level of *P* < 0.05.

### Behavioral analysis

Homozygous 1d2d-PSD-95-EGFP KI mice and WT littermate controls were generated by intercrossing heterozygous 1d2d-PSD-95-EGFP KI mice. All mice (16 mice/genotype) were male littermates and 3 to 5 months of age at the start of behavioral testing. A comprehensive battery of behavioral tests was conducted in the following sequence: general health/neurologic screen (body weight, rectal temperature), wire hang, grip strength test, light/dark transition, open field, elevated plus maze, hot plate, rotarod, social interaction (Crawley version), prepulse inhibition, Porsolt forced swim, Y-maze, 8-arm radial maze, Barnes maze, cued and contextual fear conditioning, tail suspension test, 24 h home cage monitoring (social interaction), and gait analysis, with each test separated by at least 2 days [46,47]. All the behavioral tests followed the protocols described by Takao et al. [48-50]. Behavioral data were obtained automatically by applications based on the public domain NIH Image and Image J programs and modified for each test by Tsuyoshi Miyakawa (available through Ohara & Co., Tokyo, Japan). Statistical analysis was conducted using StatView (SAS Institute, Cary, NC, USA). The data were analyzed using a two-tailed t-test, one-way or two-way analysis of variance (ANOVA), or two-way repeated measures ANOVA. The values in the graphs are expressed as the mean ± SEM. Raw data of the behavioral tests, which are not described in this paper, are shown in the mouse phenotype database (http://www.mouse-phenotype.org/).

## Abbreviations

PSD: Postsynaptic density; MAGUKs: Membrane-associated guanylate kinases; PSD-MAGUK: PSD-95 like membrane-associated guanylate kinase; SH3: Src homology-3; GK: Guanylate kinase; KI: Knockin; KO: Knockout; LTP: Long-term potentiation; LTD: Long-term depression; TARPs: Transmembrane AMPAR regulatory proteins; fEPSPs: field excitatory postsynaptic potentials; ASCF: Artificial cerebrospinal fluid; PPR: Paired-pulse ratio.

## Competing interest

The authors declare that they have no competing interests.

## Authors’ contributions

HN, YI, KK, KT, HT, SS, HS, TM and TD designed research; HN, YI, KK, TT, KN, HT, and TD performed research; KT and TM analyzed data; HN, YI, KK, KT, HT, SS, TM, YF and TD wrote the paper. All authors read and approved the final manuscript.

## Supplementary Material

Additional file 1: Figure S1 PSD morphology in WT and KI mice. Representative electron micrographs of hippocampal synapses from WT (top) and 1d2d-PSD-95-EGFP KI (bottom) mice. The PSDs were observed as thick electron-dense layers adjacent to postsynaptic membranes. No obvious differences were recognized between PSDs from WT and KI mice. Scale bar indicates 200 nm. **Figure S2.** Synaptic localization of 1d2d-PSD-95-EGFP in KI mice. Localization of 1d2d-PSD-95-EGFP was confirmed by the PSD-95 signals colocalized with an excitatory presynaptic marker, vesicular glutamate transporter-1, in hippocampal cultures from KI mice. Mouse hippocampal cultures were performed as described previously [19]. PSD-95 and vesicular glutamate transporter-1 (VGLUT1) were stained with the respective antibodies (mouse monoclonal; K28/43; 1:3000 for PSD-95, rabbit polyclonal; 1:1000 for VGLUT1; Synaptic systems), followed by Alexa Fluor-conjugated secondary antibodies. Images of the PSD-95 signals from KI mice neurons were acquired without noise filters following fluorescence excitation due to the weak signals, whereas images for WT neurons were acquired with noise filters. Scale bar, 2 μm. **Figure S3.** Comparisons of PSD protein components in the hippocampal PSD fraction of KI mice during development. Immunoblot analyses of PSD fractions from WT and KI mice at the respective developmental ages for the indicated proteins. The following amounts of WT and KI PSD proteins were analyzed per lane: AKAP75/150, 2 μg; Shank, 2 μg; Homer, 0.5 μg; mGluR5, 0.5 μg; SynGAP, 0.5 μg. There were no drastic alterations in the expression of these proteins. **Figure S4.** Robustly enhanced LTP at medial perforant path-DG synapses in KI mice. Repeated tetanus stimulation in the continuous presence of picrotoxin (100 μM) induced greater LTP at medial perforant path-DG synapses in KI (filled circles, n = 8 slices from 2 mice) mice than in WT (open circles, n = 7 slices from 3 mice) littermates at a younger age (6–8 weeks old). Black bar indicates the period of repeated high frequency stimulations. **Figure S5.** Results of the Y-maze test (I), social interaction test (II), and three-chambered social interaction test (III) of 1d2d-PSD-95-EGFP KI mice. Significant hypoactivity and decreased spontaneous alternation were observed in KI mice in a novel environment in the Y-maze test (A-D), as indicated by the number of entries (A; WT: 27.5 ± 1.5; KI: 11.6 ± 1.6; genotype effect, *F*_(1,30)_ = 51.12; *P* < 0.0001 ), total alternations (B; WT: 16.7 ± 1.2; KI: 2.25 ± 0.96; *F*_(1,30)_ = 89.90; *P* < 0.0001), alternation (C; WT: 69.2 ± 2.8%; KI: 16.4 ± 6.5%,*F*_(1,30)_ = 56.12; *P* < 0.0001), and total distance (D; WT: 2738.7 ± 131.9 cm; KI: 1438.4 ± 154.4 cm; genotype effect, *F*_(1,30)_ = 40.98; *P* < 0.0001). (II) Social interaction was normal in KI mice, as indicated by the total duration of contact (A; WT: 51.0 ± 3.7; KI: 54.1 ± 7.9; *F*_(1,14)_ = 0.122; *P* = 0.7326), number of contacts (B; WT: 52.9 ± 3.9; KI: 50.3 ± 9.0; *F*_(1,14)_ = 0.072; *P* = 0.7926), total duration of active contacts (C; WT: 16.8 ± 1.4; KI: 15.1 ± 3.2; *F*_(1,14)_ = 0.23; *P* = 0.6390), and mean duration per contact (D; WT: 0.98 ± 0.059; KI: 1.113 ± 0.126; *F*_(1,14)_ = 0.977; *P* = 0.3397). Hypoactivity was also observed in distance traveled (E; WT: 4158.3 ± 188.9 cm;KI: 2282.6 ± 231.8 cm; genotype effect, *F*_(1,14)_ = 39.35; *P* < 0.0001). (III) The three-chambered sociability and social novelty preference test was conducted as described previously [48]. (III) The results of the three-chamber social interaction test indicated that KI mice had increased anxiety and/or decreased sociability, and curiosity for novel things tended to be decreased. In both the 1st and 2nd tests, KI mice spent less time around cages, and showed a weaker tendency to spend time on the side with the stranger than WT mice. Click here for file
