# Supplementary material for: Impaired synaptic clustering of postsynaptic density proteins and altered signal transmission in hippocampal neurons, and disrupted learning behavior in PDZ1 and PDZ2 ligand binding-deficient PSD-95 knockin mice
Source: Mol Brain. 2012 Dec 26;5:43. doi: 10.1186/1756-6606-5-43 (PMC3575367; doi:10.1186/1756-6606-5-43)

Additional file 1

Figure S1

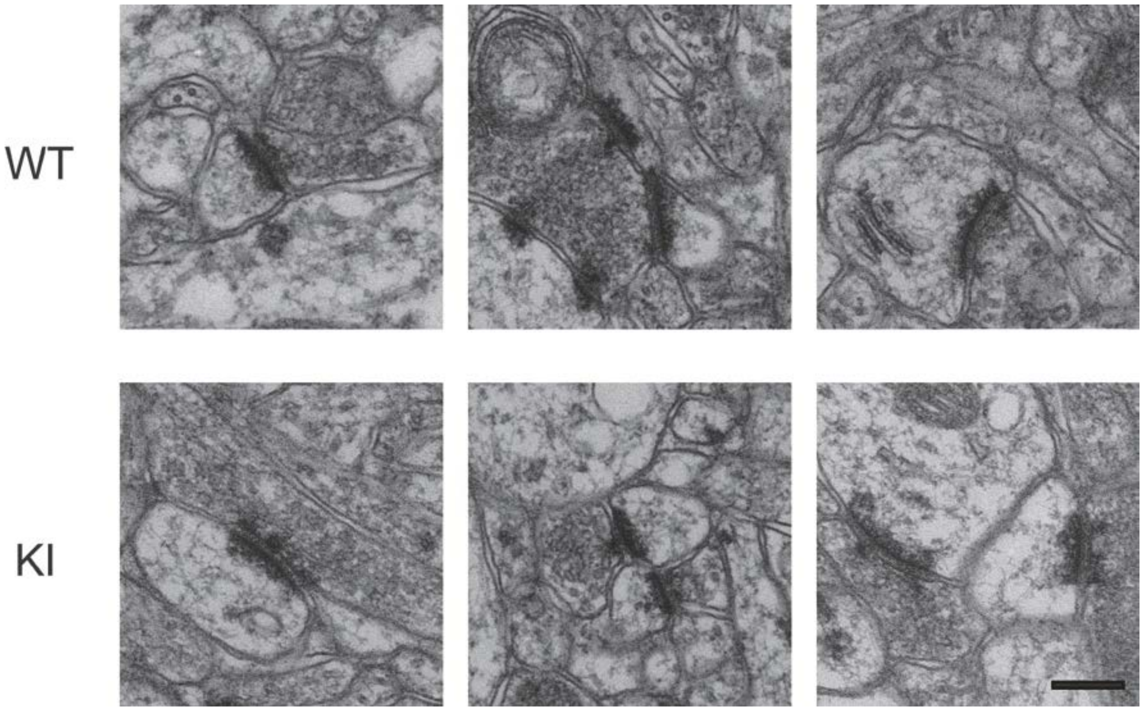

Figure S2

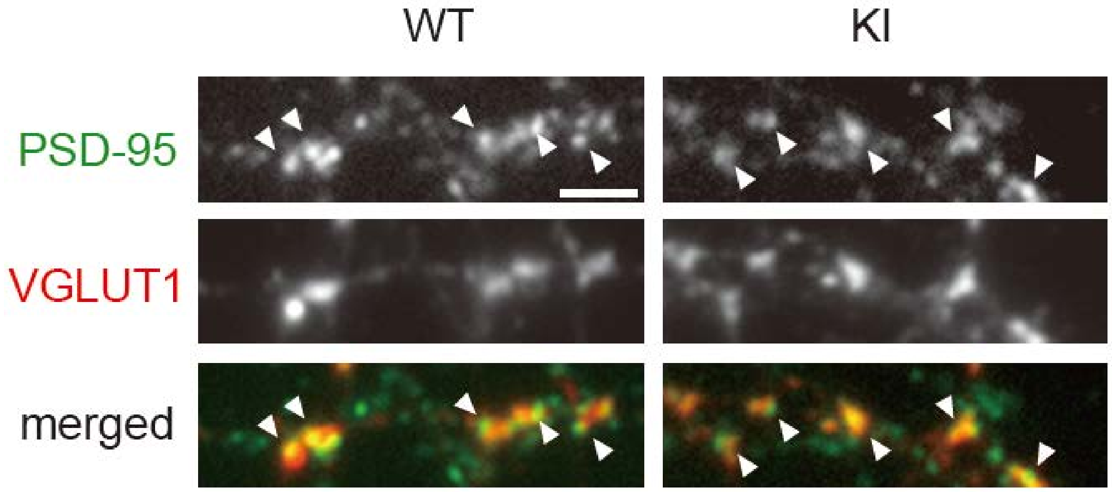

Figure S3

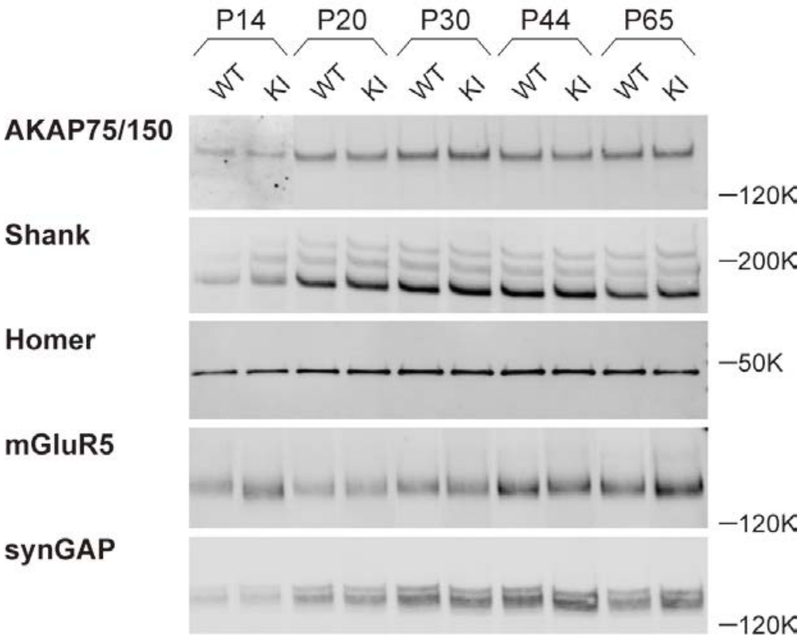
P14
P20
P30
P44
P65
WT
KI
WT
KI
WT
KI
WT
KI
WT
KI
AKAP75/150
Shank
Homer
mGluR5
synGAP
120K
200K
50K
120K
120K

Figure S4

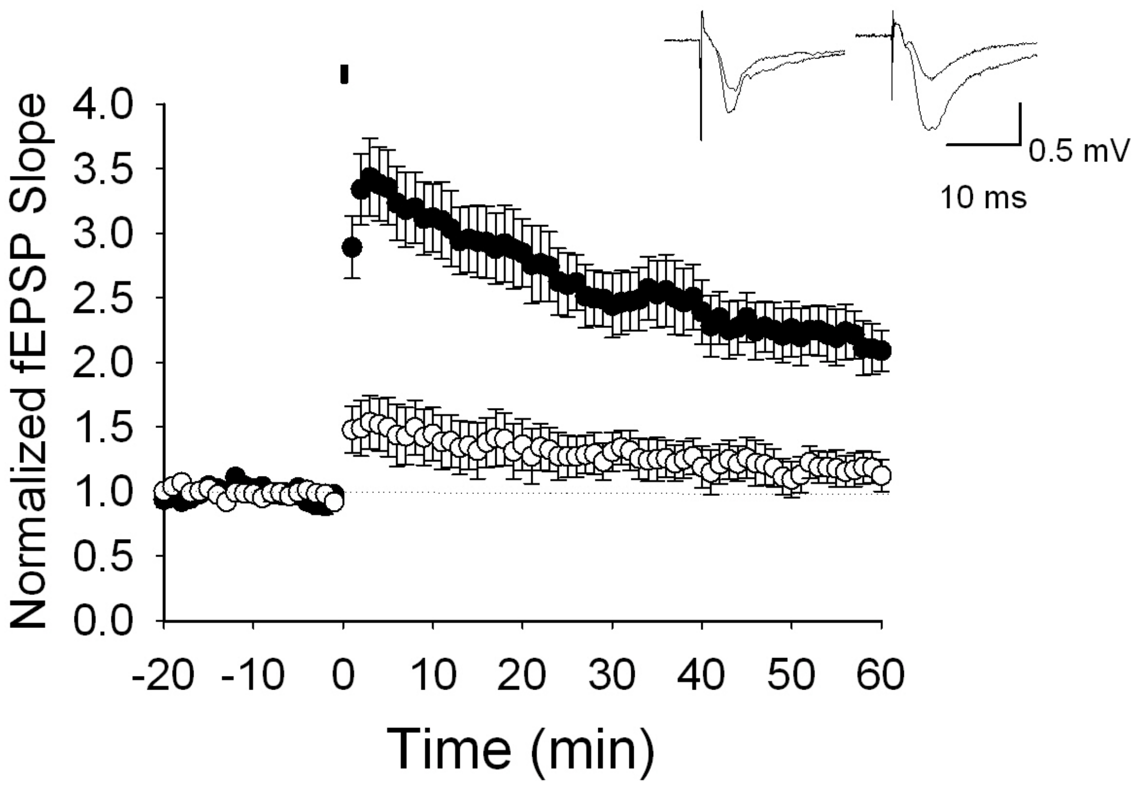

0.5 mV
10 ms
Normalized fEPSP Slope
4.0
3.5
3.0
2.5
2.0
1.5
1.0
0.5
0.0
-20
-10
0
10
20
30
40
50
60
Time (min)

Figure S5

I. Y-maze test

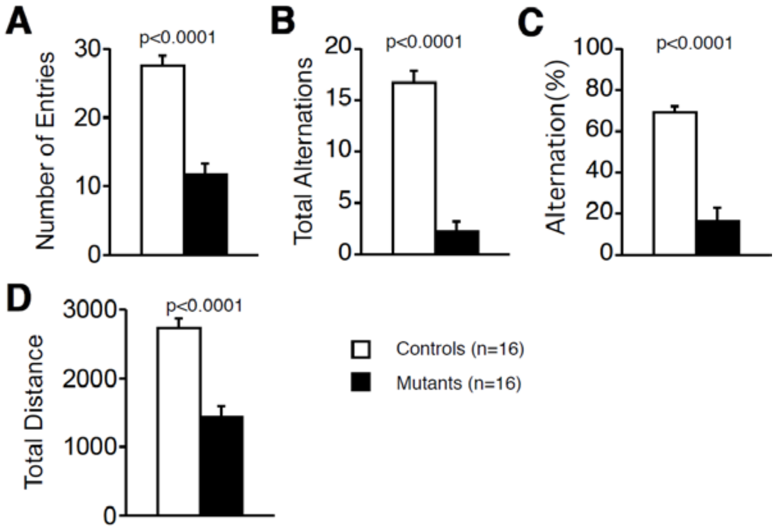

II. Social interaction test

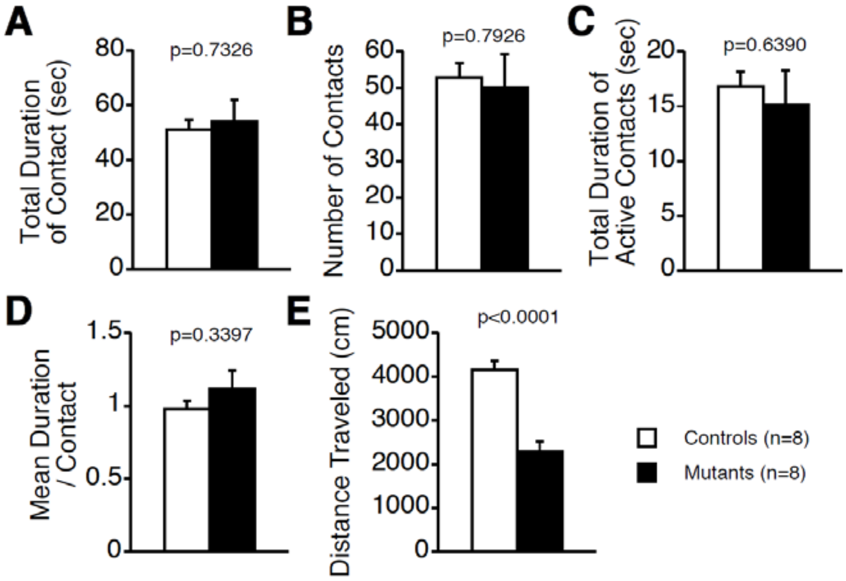

III. Three chamber social interaction

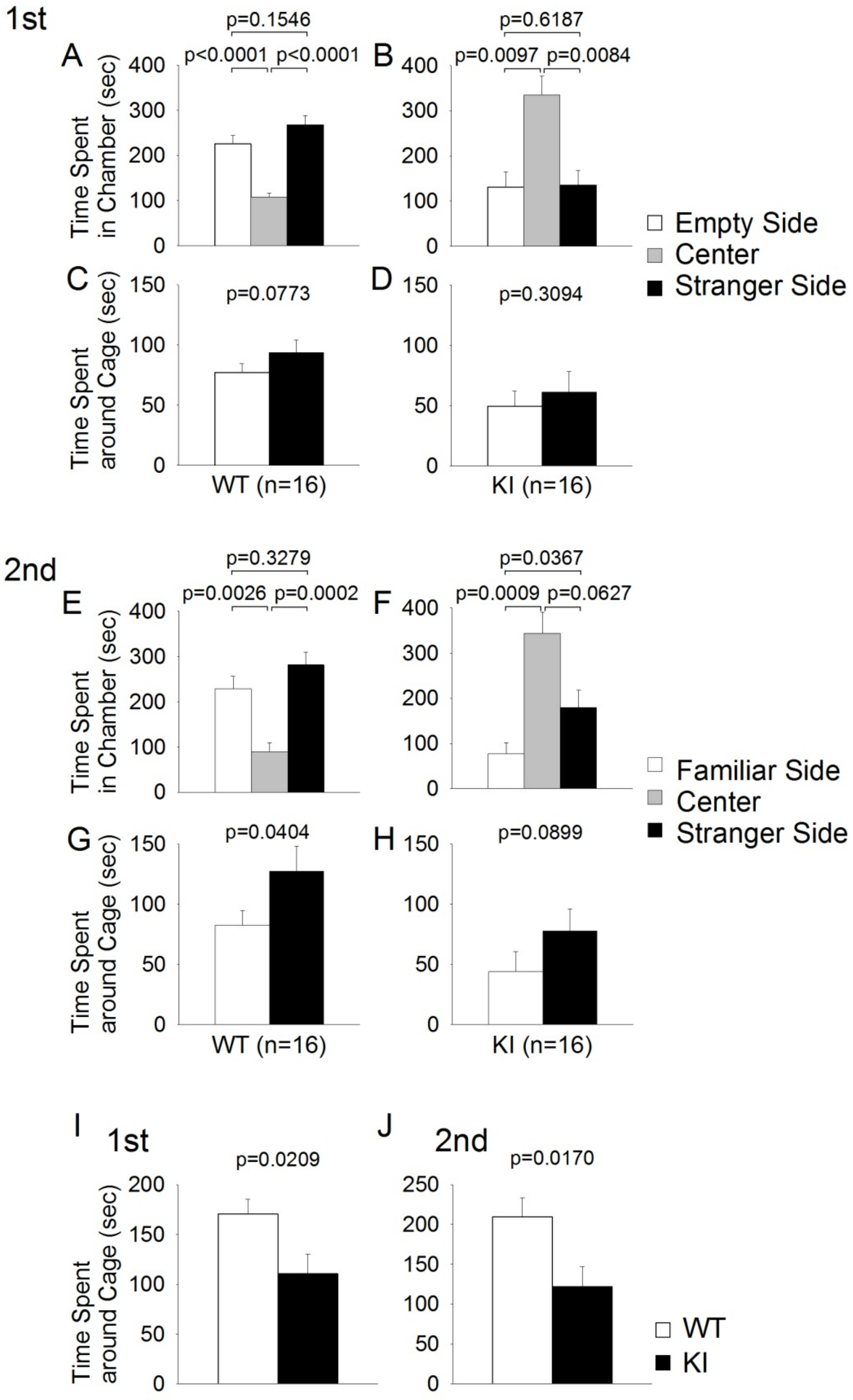

Supplement: Additional file 1: Figure S1 — PSD morphology in WT and KI mice. Representative electron micrographs of hippocampal synapses from WT (top) and 1d2d-PSD-95-EGFP KI (bottom) mice. The PSDs were observed as thick electron-dense layers adjacent to postsynaptic membranes. No obvious differences were recognized between PSDs from WT and KI mice. Scale bar indicates 200 nm. Figure S2. Synaptic localization of 1d2d-PSD-95-EGFP in KI mice. Localization of 1d2d-PSD-95-EGFP was confirmed by the PSD-95 signals colocalized with an excitatory presynaptic marker, vesicular glutamate transporter-1, in hippocampal cultures from KI mice. Mouse hippocampal cultures were performed as described previously [19]. PSD-95 and vesicular glutamate transporter-1 (VGLUT1) were stained with the respective antibodies (mouse monoclonal; K28/43; 1:3000 for PSD-95, rabbit polyclonal; 1:1000 for VGLUT1; Synaptic systems), followed by Alexa Fluor-conjugated secondary antibodies. Images of the PSD-95 signals from KI mice neurons were acquired without noise filters following fluorescence excitation due to the weak signals, whereas images for WT neurons were acquired with noise filters. Scale bar, 2 μm. Figure S3. Comparisons of PSD protein components in the hippocampal PSD fraction of KI mice during development. Immunoblot analyses of PSD fractions from WT and KI mice at the respective developmental ages for the indicated proteins. The following amounts of WT and KI PSD proteins were analyzed per lane: AKAP75/150, 2 μg; Shank, 2 μg; Homer, 0.5 μg; mGluR5, 0.5 μg; SynGAP, 0.5 μg. There were no drastic alterations in the expression of these proteins. Figure S4. Robustly enhanced LTP at medial perforant path-DG synapses in KI mice. Repeated tetanus stimulation in the continuous presence of picrotoxin (100 μM) induced greater LTP at medial perforant path-DG synapses in KI (filled circles, n = 8 slices from 2 mice) mice than in WT (open circles, n = 7 slices from 3 mice) littermates at a younger age (6–8 weeks old). Black bar indicates the [file 1756-6606-5-43-S1.pdf]
